# Preclinical validations of [^18^F]FPyPEGCBT-*c*(RGDfK): a ^18^F-labelled RGD peptide prepared by ligation of 2-cyanobenzothiazole and 1,2-aminothiol to image angiogenesis

**DOI:** 10.1186/s41181-016-0019-z

**Published:** 2016-10-25

**Authors:** Didier J. Colin, James A. H. Inkster, Stéphane Germain, Yann Seimbille

**Affiliations:** 1 MicroPET/SPECT/CT Imaging Laboratory, Centre for BioMedical Imaging (CIBM), University Hospital of Geneva, 1211 Geneva, Switzerland; 2 Cyclotron Unit, University Hospital of Geneva, 1211 Geneva, Switzerland; 3 TRIUMF, Life Sciences Division, 4004 Wesbrook Mall, Vancouver, BC V6T 2A3 Canada

**Keywords:** Angiogenesis, Integrins, RGD peptide, Fluorine-18, MicroPET imaging

## Abstract

**Background:**

α_V_β_3_, α_V_β_5_ and α_5_β_1_ integrins are known to be involved in carcinogenesis and are overexpressed in many types of tumours compared to healthy tissues; thereby they have been selected as promising therapeutic targets. Positron emission tomography (PET) is providing a unique non-invasive screening assay to discriminate which patient is more prone to benefit from antiangiogenic therapies, and extensive research has been carried out to develop a clinical radiopharmaceutical that binds specifically to integrin receptors. We recently reported the synthesis of a new ^18^F-labelled RGD peptide prepared by 2-cyanobenzothiazole (CBT)/1,2-aminothiol conjugation. This study aims at characterising the preclinical biologic properties of this new tumour-targeting ligand, named [^18^F]FPyPEGCBT-*c*(RGDfK).

The in vitro binding properties of [^18^F]FPyPEGCBT-*c*(RGDfK) were analysed by standard binding assay in U-87 MG and SKOV-3 cancer models and its selectivity towards integrins by siRNA depletions. Its preclinical potential was studied in mice bearing subcutaneous tumours by ex vivo biodistribution studies and in vivo microPET/CT imaging.

**Results:**

In vitro, FPyPEGCBT-*c*(RGDfK) efficiently bound RGD-recognising integrins as compared to a control *c*(RGDfV) peptide (IC_50_ = 30.8 × 10^−7^ M vs. 6.0 × 10^−7^ M). [^18^F]FPyPEGCBT-*c*(RGDfK) cell uptake was mediated by an active transport through binding to α_V_, β_3_ and β_5_ but not to β_1_ subunits. In vivo, this new tracer demonstrated specific tumour uptake with %ID/g of 2.9 and 2.4 in U-87 MG and SKOV-3 tumours 1 h post injection. Tumour-to-muscle ratios of 4 were obtained 1 h after intravenous administration of the tracer allowing good visualisation of the tumours. However, unfavourable background accumulation and high hepatobiliary excretion were observed.

**Conclusion:**

[^18^F]FPyPEGCBT-*c*(RGDfK) specifically detects tumours expressing RGD-recognising integrin receptors in preclinical studies. Further optimisation of this radioligand may yield candidates with improved imaging properties and would warrant the further use of this efficient labelling technique for alternative targeting vectors.

**Electronic supplementary material:**

The online version of this article (doi:10.1186/s41181-016-0019-z) contains supplementary material, which is available to authorized users.

## Background

Positron Emission Tomography (PET) is a sensitive nuclear imaging method to diagnose and stage cancers, particularly when images are co-registered with Computed Tomography (CT) or Magnetic Resonance Imaging (MRI) (Gambhir 2002; Yankeelov et al. 2014). Diagnosis of patients suspected of having cancer is commonly investigated by PET/CT scans with 2-deoxy-2-[^18^F]fluoro-d-glucose ([^18^F]FDG) (Fletcher et al. 2008). Furthermore, 3'-deoxy-3'-[^18^F]fluorothymidine ([^18^F]FLT), a positron emitting thymidine derivative, is often used to monitor cancer response towards chemotherapy (Tehrani and Shields 2013). Both small molecule tracers have shown diagnostic potential and efficacy in predicting tumour response to treatment, but only after initiation of the therapeutic regimen. Moreover, their organ uptakes are mediated by high tissue metabolism and proliferation, respectively, which impairs their specificity towards tumour tissue. Thus, identification of PET tracers targeting specific proteins which are solely overexpressed in cancer tissues (e.g. cell membrane receptors) (Eberle and Mild 2009) could greatly facilitate the deployment of personalised medicine strategies (Doroshow and Kummar 2014).

Several cell membrane receptors are known to be highly expressed in cancer tissues. Among them, integrin receptors are of particular interest since they are implicated in angiogenesis, tumour growth and metastasis. They are also overexpressed in endothelial cells of the tumor vasculature where they promote cell migration (Avraamides et al. 2008; Desgrosellier and Cheresh 2010). The Arginine-Glycine-Aspartic acid (RGD)-recognising α_V_β_3_, α_V_β_5_ and α_5_β_1_ heterodimeric transmembrane receptors have been selected as the most attractive targets among the integrin receptors for antiangiogenic therapy and diagnosis, as recently and exhaustively reviewed by Danhier et al. (Danhier et al. 2012). RGD-recognising integrins are involved in other physiopathological processes implicating angiogenesis and/or inflammation (e.g. atherosclerosis), which further highlights the importance of these targets for the development of non-invasive imaging probes (Antonov et al. 2004).

A number of RGD-derived peptide tracers for integrin-based PET imaging have already been described. Among them, those labelled with ^64^Cu and ^68^Ga radionuclides have shown promising preclinical results (Dumont et al. 2011; Pohle et al. 2012). However, to date only [^68^Ga]NOTA-*c*(RGDyK) has been evaluated clinically (Jeong et al. 2008; Kim et al. 2012). More ^18^F-labelled RGD peptides have reached clinical trials (Cai and Conti 2013; Haubner et al. 2014a), in large part due to the attractive nuclear properties of ^18^F and its pervasive availability at most PET centres. Among the most potent RGD peptides is the glycosylated cyclic RGD analogue, [^18^F]galacto-*c*(RGDfK), which has shown specific binding to integrin-expressing tumours both in preclinical and clinical settings (Haubner et al. 2001; Haubner et al. 2005; Beer et al. 2006; Haubner et al. 2004). An attempt to optimise the radiosynthesis and in vivo biodistribution of such sugar-tethered RGD radiopeptides was recently investigated, and efforts yielded a potent maltose-derived tracer (Maschauer et al. 2014). In parallel, in vivo behaviours of RGD tracers bearing amphiphilic polyethylene glycol (PEG) chains (Harris and Chess 2003), such as the PEGylated [^18^F]Fluciclatide analogue ([^18^F]-AH111585), were also studied (Tomasi et al. 2011; Kenny et al. 2008; Battle et al. 2011). Another approach is to use petides containing two or more integrin-recognising units to increase the affinity and the bioavailability. Dimers of *c*(RGDyK) were labelled with *N*-succinimidyl 4-[^18^F]fluorobenzoate ([^18^F]SFB) and 4-nitrophenyl 2-[^18^F]fluoropropionate ([^18^F]NFP) to yield [^18^F]FRGD2 and [^18^F]FPPRGD2, respectively (Chen et al. 2004a; Zhang et al. 2006; Wu et al. 2007a; Choyke 2011; Mittra et al. 2011; Liu et al. 2010). They are both good candidate tracers to image angiogenesis and are currently under clinical investigations.

**Fig. 1:**
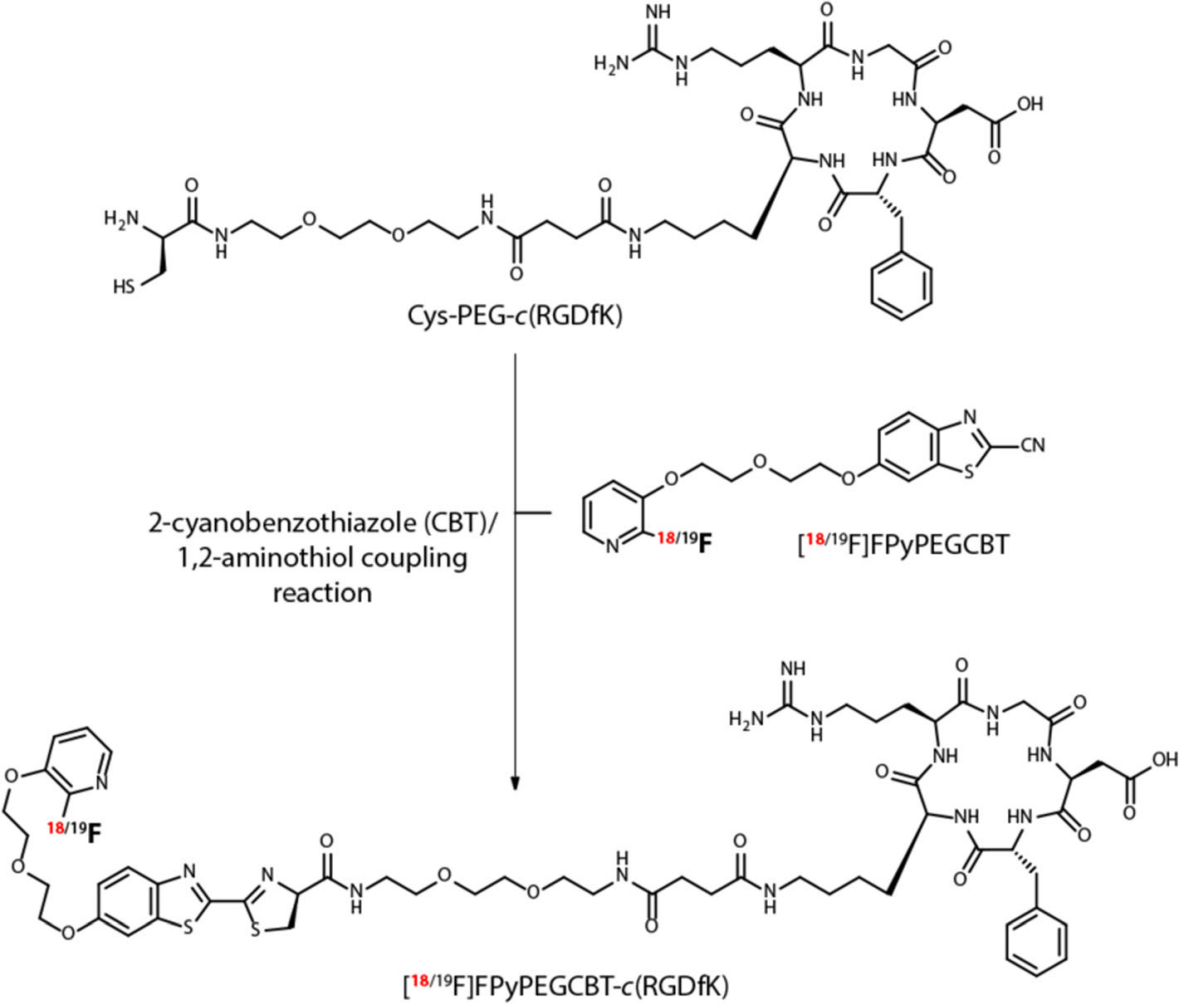
Synthesis of [^18,19^F]FPyPEGCBT-*c*(RGDfK)

In light of these encouraging results, we recently synthesised a new *c*(RGDfK)-based PET tracer via conjugation of a ^18^F-labelled PEGylated cyanobenzothiazole prosthetic group ([^18^F]FPyPEGCBT) with a *c*(RGDfK) peptide containing a cysteine residue at the terminus of the PEG chain (Fig. 1) (Inkster et al. 2015). This bioconjugation reaction was chosen since it adheres to all “click chemistry” criteria and it has previously demonstrated great potential to label sensitive biomolecules (Kolb et al. 2001; Jeon et al. 2012; Kettenbach et al. 2014). We report herein the preclinical biological characteristics of [^18^F]FPyPEGCBT-*c*(RGDfK). In vitro binding affinity and selectivity of [^18^F]FPyPEGCBT-*c*(RGDfK) towards α_V_β_3_, α_V_β_5_ and α_5_β_1_ integrins were assessed in U-87 MG and SKOV-3 relevant glioblastoma and ovarian cancer cell lines. Its potential as a molecular imaging agent was further studied in mice bearing the same tumour models by ex vivo biodistribution assays and in vivo microPET/CT imaging.

## Methods

### Chemicals

All reagents were obtained from Sigma-Aldrich (Buchs, Switzerland) unless specified.

### Radiochemistry

[^18^F]FPyPEGCBT-*c*(RGDfK) was prepared via the reported method (Inkster et al. 2015). In summary, ^18^F prosthetic group [^18^F]FPyPEGCBT was prepared by way of nucleophilic aromatic [^18^F]fluorination of its 2-trimethylammonium pyridine precursor and partially purified by solid-phase extraction on molecularly imprinted polymer sorbent (AffiniMIP®). [^18^F]FPyPEGCBT was then coupled to cysteine-modified *c*(RGDfK) in DMF over 30 minutes at 43 °C. [^18^F]FPyPEGCBT-*c*(RGDfK) was purified by High Performance Liquid Chromatography (HPLC) and formulated in 10 % EtOH in isotonic saline for preclinical use. The entire synthesis was completed on a single automated synthesis unit (GE Healthcare, Ecublens, Switzerland) over 124–132 min.

### Cell lines

The U-87 MG glioblastoma and SKOV-3 ovarian cancer cell lines (ECACC, Salisbury, UK) were cultured at 37 °C and 5 % CO_2_ in Dulbecco’s Modified Eagle Medium (DMEM; Life Technologies, Zug, Switzerland) supplemented with 10 % (v/v) fetal calf serum (Life Technologies).

### siRNA transfection

siRNAs targeting human α_V_, β_1_, β_3_ or β_5_ integrin subunits were synthesised by MWG (Ebersberg, Germany). siRNA sequences used were: for α_V_, sense 5′-UCCAUUCAUGUACUUUUCC-3′; β_1_, sense 5′-AUGUAACCAACCGUAGCA-3′; β_3_, sense 5′-CAAGCCUGUGUCACCAUAC-3′; β_5_, sense 5′-GCUCGCAGGUCUCAACAUA-3′. An siRNA targeting luciferase was used as a non-specific control, sense 5′-CGUACGCGGAAUACUUCGA-3′. 48 h prior to an experiment, U-87 MG (4 × 10^4^ cells per well) and SKOV-3 (1 × 10^4^ cells per well) cells were reverse transfected in 96-well plates with a final concentration of 50 nM of indicated siRNAs using RNAiMAX (Life Technologies) according to the manufacturer’s protocol.

### Immunoblotting

Protein extracts were prepared in a RIPA buffer (50 mM Tris, 150 mM NaCl, 0.1 % SDS, 0.5 % Na deoxycholate, 1 % Igepal CA630, 2 mM EDTA, 50 mM NaF, pH 8) containing a cocktail of protease inhibitors (Roche, Rotkreuz, Switzerland) and titrated using the DC Protein Assay (Biorad, Cressier, Switzerland). Five to 10 μg of protein extracts were separated by SDS-PAGE and transferred onto nitrocellulose membranes (Amersham, GE Healthcare, Glattbrugg, Switzerland). Membranes were blocked for 1 h at room temperature, incubated overnight at 4 °C with primary antibodies, incubated for 1 h with secondary antibodies, developed using standard ECL protocol and analysed using a Chemidoc MP (Biorad). The primary antibodies raised against α_V_ (611012), β_1_ (610467) and β_3_ (611140) integrins were from BD (Allschwil, Switzerland), against β_5_ integrin (3629S) from Cell Signalling (Berverly, MA, USA) and Actin (A2066) from Sigma-Aldrich. HRP-conjugated secondary antibodies were obtained from Biorad.

### Cell binding assay

In vitro integrin-binding of FPyPEGCBT-*c*(RGDfK) was evaluated using a standard displacement assay of [^125^I]echistatin. U-87 MG and SKOV-3 cells were seeded in 96-well plates 24 h prior to the experiment (U-87 MG, 6 × 10^4^ cells per well; SKOV-3, 3 × 10^4^ cells per well). On the day of the experiment, the cells were washed twice with binding buffer (20 mM Tris pH 7.4, 150 mM NaCl, 2 mM CaCl_2_, 1 mM MgCl_2_, 1 mM MnCl_2_, 0.1 % BSA), then co-incubated for 1 h at 37 °C in binding buffer with 370 Bq/well [^125^I]echistatin (Perkin Elmer, Schwerzenbach, Switzerland) and a concentration range (10^−9^ to 10^−4^ M) of FPyPEGCBT-*c*(RGDfK) or *c*(RGDfV) (PeptaNova, Sandhausen, Germany) as a reference peptide. Cells were then washed three times with binding buffer and lysed in 1 M NaOH. The cell-associated radioactivity was measured in a Wizard^2^ 2470 γ-counter (Perkin Elmer). The IC_50_ (Inhibitory Concentration of 50 %) values were calculated by fitting the data by nonlinear regression using GraphPad Prism (GraphPad software, La Jolla, CA, USA).

### Cell uptake assay

U-87 MG (6 × 10^4^ cells per well) and SKOV-3 (3 × 10^4^ cells per well) cells were seeded in 96-well plates 24 h prior to the experiment. On the day of the experiment, cells were washed twice with binding buffer (20 mM Tris pH 7.4, 150 mM NaCl, 2 mM CaCl_2_, 1 mM MgCl_2_, 1 mM MnCl_2_, 0.1 % BSA) then incubated for 15 to 120 min with 100 kBq/mL [^18^F]FPyPEGCBT-*c*(RGDfK) in binding buffer at 37 or 4 °C. Cells were also co-treated with 10^−4^ M *c*(RGDfV) for 1 h. [^18^F]FPyPEGCBT-*c*(RGDfK) uptake in cells with siRNA-mediated depletions of specific integrin subunits was also assessed. After indicated uptake times, cells were washed three times with binding buffer and lysed in 1 M NaOH. The cell-associated radioactivity was measured in a Wizard^2^ 2470 γ-counter (Perkin Elmer).

### Animal models

All animal experiments were performed in compliance with the current Swiss animal protecting laws and protocols of the local authorities (approval G36/3528). Six to eight weeks old female BALB/c nude mice (Harlan, Horst, The Netherlands) were injected subcutaneously into the right flank with 1 × 10^6^ U-87 MG or SKOV-3 cancer cells in 200 μL of a matrix containing 30 % of DMEM and 70 % of HyStem-C hydrogel (ESI BIO, Alameda, CA, USA). After two weeks, when tumour volumes were approximately 500 mm^3^, the animals were used for microPET/CT and/or biodistribution studies.

### Biodistribution study

Mice were anesthetised with 2 % isoflurane and were injected retro-orbitally in the venous sinus with 3–5 MBq of [^18^F]FPyPEGCBT-*c*(RGDfK). Mice were then left awake during the uptake times of 30, 60 or 120 min. For the blocking experiments, mice were injected intravenously with 20 mg/kg of *c*(RGDfV) 5 min prior to the tracer. Mice were then anesthetised, blood was collected by intracardiac puncture and the animals were sacrificed. Indicated organs and tumours were collected, weighed, and the radioactivity quantified in a Wizard^2^ 2470 γ-counter. Data were expressed as percentages of the injected dose per gram (%ID/g).

### MicroPET/CT imaging

Injections of [^18^F]FPyPEGCBT-*c*(RGDfK) to anesthetised mice were conducted as described above. Mice were injected intraperitoneally with 700 μL of 132 mg/mL meglumine ioxitalamate (Telebrix, 6 % m/v iodide, Guerbet AG, Zürich, Switzerland) to allow abdominal organs discrimination and subjected to CT scans in a Triumph microPET/SPECT/CT system (Trifoil, Chatsworth, CA, USA). Images were obtained at 80 kVp, 160 μA, and 1024 projections were acquired during the 360° rotation with a field of view of 71.3 mm (1.7× magnification). After 20 min of uptake, PET scans were started for a total duration of 110 min allowing the reconstructions of PET frames of 30 ± 10 min, 60 ± 10 min and 120 ± 10 min of uptake. PET scans were reconstructed with the LabPET software using an OSEM3D (20 iterations) algorithm and images were calibrated in Bq/mL by scanning a phantom cylinder. The Triumph XO software, which uses a back-projection engine, was used to reconstruct the CT scans with a reconstruction matrix of 512 and a voxel size of 0.135 mm. Reconstructed CTs were then co-registered with the PET scans using the plugin Vivid (Trifoil) for Amira (FEI, Hillsboro, OR, USA) and exported as dicom files for mice anatomy visualisation. The software Osirix (Pixmeo, Geneva, Switzerland) was used to quantitatively analyse the datasets and generate pictures. PET series were converted to display Standardised Uptake Values (SUV) adjusted to the body weight of the animals and merged with CT sets. Regions of interest (ROI) were drawn on contiguous slides according to CT scans. ROIs were subsequently computed to 3D volumes allowing the quantification of the [^18^F]FPyPEGCBT-*c*(RGDfK) uptake in the different organs.

### Metabolic assay

Fractions of blood collected during biodistribution studies were used to assess metabolic stability of [^18^F]FPyPEGCBT-*c*(RGDfK). Metabolic activity was quenched (v/v) with a solution of 20 % MeCN in PBS (100 mM, pH 7.4). Blood samples were centrifuged at 4 °C (2000 × g, 8 min) and the serum was removed. Serum proteins were precipitated with MeCN (1:1) and centrifuged at 4 °C again (21380 × g, 8 min). Supernatants were removed from pellets, and the activity associated with both was measured. In this fashion, the extraction efficiency of this step was estimated to be 91.0 % ± 4.8 (*n* = 18). For each sample, an aliquot of supernatant (2 μL) was spotted onto silica gel plates and eluted with 4:6 MeOH-10 % (w/w) *aqueous* ammonium acetate. Phosphor imaging screens were exposed to dried TLC plates for 10–25 min and analysed by autoradiography using a Cyclone plus and the built-in Optiquant software (Perkin Elmer).

### Figures and statistics

Statistical analyses and regressions were conducted using Graphpad prism. Differences between samples were assessed using one-way ANOVA statistical test. Figures were generated using Microsoft Excel, Graphpad Prism, Adobe Photoshop and Adobe Illustrator.

## Results

### Radiochemistry

The ^18^F peptide tracer FPyPEGCBT-*c*(RGDfK) (276–641 MBq) was prepared in yields of 7 % ± 1 (*n* = 9) from end-of-bombardment over 124–132 min. Radiochemical purity, determined by HPLC, was above 95 % and the specific activity was estimated to be 4–12 GBq μmol^−1^.

### In vitro cell binding assay

**Fig. 2:**
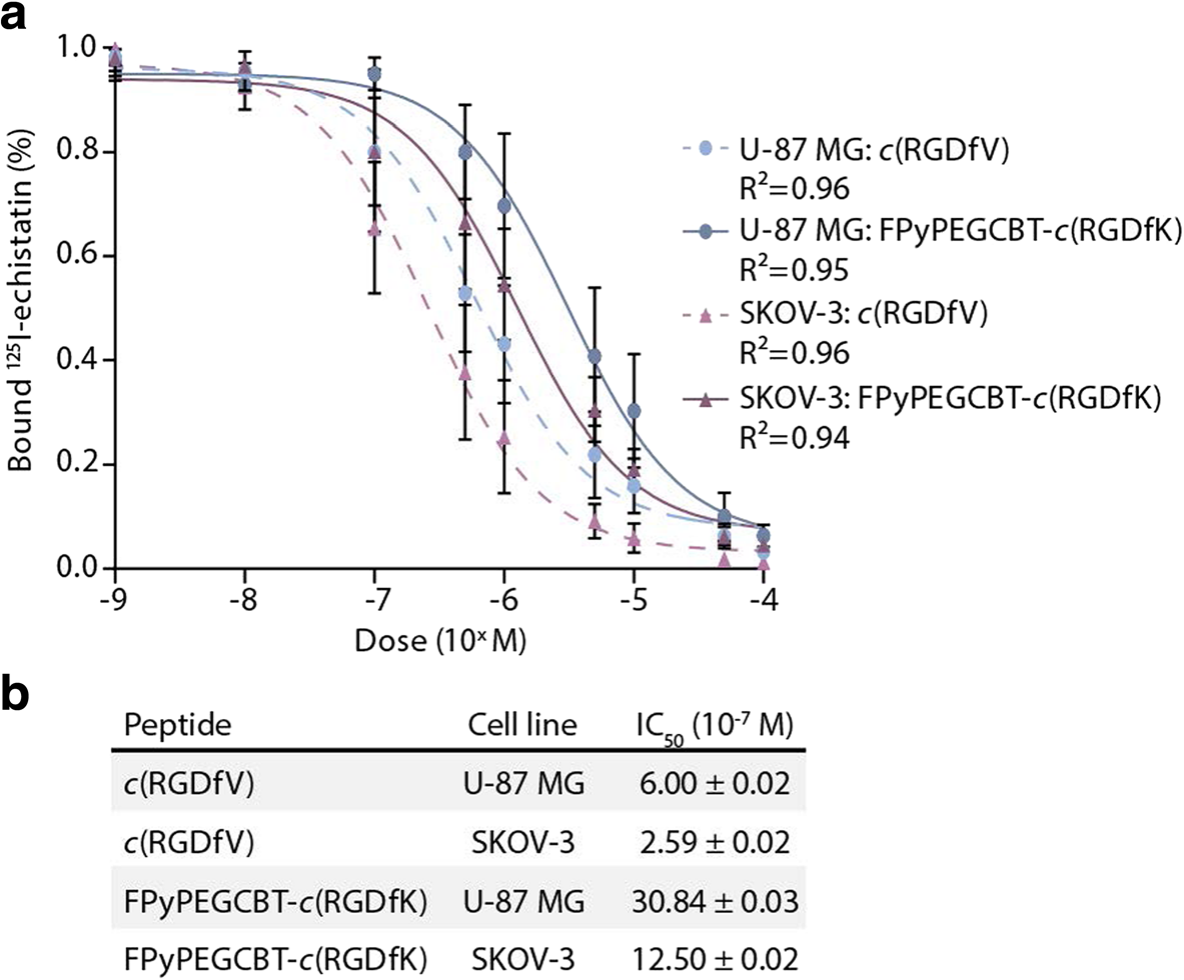
Displacement of [^125^I]echistatin by FPyPEGCBT-*c*(RGDfK). U-87 MG and SKOV-3 cancer cells were co-incubated for 1 h in 96-well plates with 370 Bq/well [^125^I]echistatin and a concentration range (10^−9^ to 10^−4^ M) of FPyPEGCBT-*c*(RGDfK) or *c*(RGDfV). After lysis, the cell-associated radioactivity was measured in a γ-counter. Means ± SD of three independent experiments conducted in quadruplicate well were fitted by nonlinear regression using GraphPad Prism and R^2^ fitting correlation coefficients are shown (**a**). IC_50_ values ± SD are also shown (**b**)

The binding affinity of FPyPEGCBT-*c*(RGDfK) was evaluated using a standard in vitro binding assay on U-87 MG and SKOV-3 cancer cells expressing α_V_β_3_, α_V_β_5_ and α_5_β_1_ integrins and compared to a reference peptide *c*(RGDfV) (Fig. 2). FPyPEGCBT-*c*(RGDfK) inhibited the binding of [^125^I]echistatin in both models in a dose-dependent manner. The IC_50_ values of FPyPEGCBT-*c*(RGDfK) were 30.84 ± 0.02 and 12.50 ± 0.02 × 10^−7^ M in U-87 MG and SKOV-3 models respectively, as compared to 6.00 ± 0.02 and 2.59 ± 0.02 × 10^−7^ M for *c*(RGDfV). IC_50_ values of FPyPEGCBT-*c*(RGDfK) are only five times higher than those of the control peptide in both cell lines. It is confirming that the substitution on the lysine residue has little effect on the binding affinity of cyclic RGD peptides.

### Cell uptake of [^18^F]FPyPEGCBT-*c*(RGDfK)

**Fig. 3:**
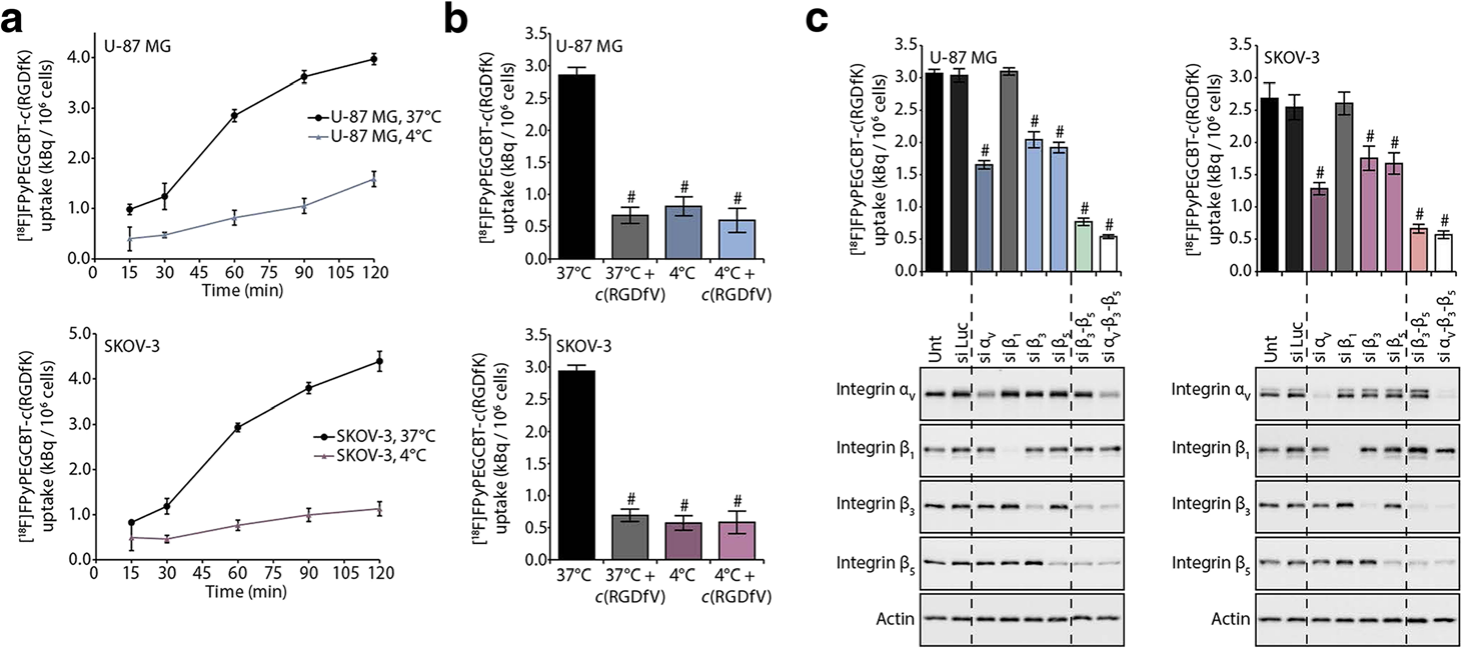
Uptake of [^18^F]FPyPEGCBT-*c*(RGDfK) in cancer cells. U-87 MG and SKOV-3 cells were incubated with 100 kBq/mL [^18^F]FPyPEGCBT-*c*(RGDfK) for 15 to 120 min at 37 or 4 °C (**a**). Cells were also co-treated with 10^−4^ M *c*(RGDfV) for 1 h (**b**). Uptake of [^18^F]FPyPEGCBT-*c*(RGDfK) was also evaluated after 1 h of incubation with cells with selective siRNA-mediated knocked down of α_V_, β_1_, β_3_ or β_5_ integrin subunits and indicated combinations (**c**). Depletions were verified by immunoblotting using specific antibodies as compared to untransfected cells (Unt) and to an irrelevant depletion of luciferase (si Luc). After incubation, cells were lysed and the cell-associated radioactivity was measured in a γ-counter. Data are means ± SD of at least three independent experiments performed in quadruplicate well. Statistical differences were analysed with the one-way ANOVA test followed by Dunnett’s post hoc test as compared to 37 °C controls (**b**) or to si Luc (**c**); #*p* < 0.001

Incubation of U-87 MG or SKOV-3 cells with [^18^F]FPyPEGCBT-*c*(RGDfK) showed that this tracer was taken up in a time-dependent manner at a physiological temperature of 37 °C (Fig. 3). The influx of the peptide was inhibited by five to six fold when incubation was performed at 4 °C for 60 min. This difference demonstrated that [^18^F]FPyPEGCBT-*c*(RGDfK) was primarily taken up by active transporters (Fig. 3a). Co-treatment with an excess of *c*(RGDfV) blocked by five to six times the accumulation of [^18^F]FPyPEGCBT-*c*(RGDfK) in cells, to a level corresponding to its passive diffusion (as measured at 4 °C). Overall, our data confirm that the cellular uptake of this peptide is effectively mediated by RGD-recognising integrins (Fig. 3b).

Subsequently, the binding specificity of [^18^F]FPyPEGCBT-*c*(RGDfK) to integrin subunits was assessed by selective knock down of α_V_, β_1_, β_3_ or β_5_ in cell uptake assays (Fig. 3c). The siRNA-mediated depletions of each protein in U-87 MG and SKOV-3 cells were highly reproducible and no significant interference or feedback on the expression of non-targeted integrins was observable (Additional file 1: Figure S1). Densitometry analyses showed that the efficacies of knock down were good in U-87 MG (between 66 and 98 %) and even better in SKOV-3 cells (between 75 and 97 %). Validity of these depletion models was further confirmed by testing their functionality in [^125^I]echistatin uptake assays. These showed that lowering α_V_, β_1_, β_3_ or β_5_ expressions highly affected the echistatin uptake (Additional file 1: Figure S2). [^18^F]FPyPEGCBT-*c*(RGDfK) uptake studies in integrin subunit depleted cells demonstrated that α_V_, β_3_ and β_5_ are highly involved in the influx of this radio-peptide since their knock down strongly interfered with [^18^F]FPyPEGCBT-*c*(RGDfK) accumulation in both cell lines (Fig. 3c). However, depletion of subunit β_1_ did not interfere with the tracer uptake.

### Ex vivo biodistribution in tumour-bearing mice

**Fig. 4:**
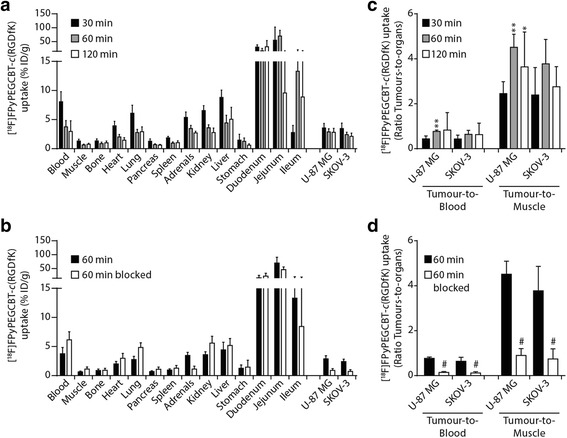
Biodistribution of [^18^F]FPyPEGCBT-*c*(RGDfK) in nude mice bearing U-87 MG or SKOV-3 subcutaneous tumours. Mice intravenously injected with 3–5 MBq [^18^F]FPyPEGCBT-*c*(RGDfK) were sacrificed and indicated organs were collected after uptake times of 30, 60 or 120 min (**a**). For the blocking experiments, mice were injected intravenously with 20 mg/kg of *c*(RGDfV) 5 min prior to [^18^F]FPyPEGCBT-*c*(RGDfK) injection and indicated organs were collected after 60 min of uptake (**b**). [^18^F]FPyPEGCBT-*c*(RGDfK) uptake in the indicated organs was quantified in a γ-counter and expressed as percentages of the injected dose per gram (%ID/g) (**a**, **b**). Tumours-to-blood and Tumours-to-muscle %ID/g ratios were also determined for the uptake time course of [^18^F]FPyPEGCBT-*c*(RGDfK) (**c**) and the blocked experiment (**d**). Data are means ± SD (*n* ≥ 7) and statistical differences were analysed with the one-way ANOVA test followed by Dunnett’s post hoc test as compared with 0.5 h uptake time (**c**) or with non-blocked 1 h uptake time (**d**); *p < 0.05, **p < 0.01, #*p* < 0.001

The biodistribution profile of [^18^F]FPyPEGCBT-*c*(RGDfK) was assessed in mice bearing subcutaneous U-87 MG glioblastoma or SKOV-3 ovarian human tumours (Table 1, Fig. 4a, c, and Additional file 1: Table S1). The uptake of the labelled peptide decreased over time in both models with %ID/g ranging from 3.6 ± 0.8 to 2.8 ± 0.7 in U-87 MG and 3.5 ± 0.9 to 2.1 ± 0.6 in SKOV-3 tumours. Retention of the tracer in the blood remained high (3.8 ± 1.1 %ID/g after 1 h) leading to tumour-to-blood ratios which were not optimal (0.8 ± 0.1 for U-87 MG and 0.6 ± 0.2 for SKOV-3 at 60 min post injection). The in vivo stability study showed that blood contained only intact peptide tracer at all time points (Additional file 1: Figure S4). High tumour-to-muscle ratios of 4.5 ± 0.6 for U-87 MG and 3.8 ± 1.1 for SKOV-3 were observed at 1 h post injection (Fig. 4c). In addition, the uptake in many non-targeted organs significantly decreased over time, which is favourable for a good visualisation of the tumours (see Additional file 1: Table S1 for statistical analyses). Organs implicated in the excretory pathways accumulated the peptide tracer as expected, and the %ID/g in the kidneys and liver decreased in a time-dependent manner. Nevertheless, the uptake in the intestine remained high over the time and highlighted the importance of the hepatobiliary pathway in the clearance of [^18^F]FPyPEGCBT-*c*(RGDfK). Co-injection of *c*(RGDfV) significantly blocked by three to four times the tumour uptake of [^18^F]FPyPEGCBT-*c*(RGDfK) (from 2.9 to 0.9 %ID/g in U-87 MG and 2.4 to 0.7 in SKOV-3) showing the specificity of its uptake (Table 1, Fig. 4b, d, and Additional file 1: Table S1). No significant differences were found in other organs, except the adrenals, which are known to express moderate levels of RGD-recognising integrins.

**Table 1:** Biodistribution data of [^18^F]FPyPEGCBT-*c*(RGDfK) in nude mice bearing U-87 MG or SKOV-3 subcutaneous tumours^*a*^

Organ	30 min	60 min	60 min blocking	120 min
Blood	8.08 ± 1.72	3.76 ± 1.08	6.14 ± 1.39	2.96 ± 1.83
Muscle	1.32 ± 0.32	0.64 ± 0.14	1.12 ± 0.38	0.77 ± 0.16
Bone	1.34 ± 0.34	0.88 ± 0.25	0.91 ± 0.35	1.02 ± 0.30
Heart	3.92 ± 0.78	2.01 ± 0.43	2.99 ± 0.82	1.45 ± 0.45
Lung	6.10 ± 1.39	2.77 ± 0.55	4.83 ± 0.82	2.93 ± 0.82
Pancreas	1.29 ± 0.40	0.71 ± 0.13	1.09 ± 0.39	0.63 ± 0.12
Spleen	1.87 ± 0.25	0.96 ± 0.21	1.28 ± 0.46	1.03 ± 0.34
Adrenals	5.37 ± 0.94	3.47 ± 0.53	1.13 ± 0.51	2.71 ± 0.29
Kidney	6.56 ± 0.85	3.61 ± 0.49	5.59 ± 1.17	2.77 ± 0.77
Liver	8.82 ± 1.25	4.43 ± 1.31	5.19 ± 1.19	5.04 ± 2.07
Stomach	1.48 ± 1.00	1.26 ± 0.58	1.45 ± 1.21	0.60 ± 0.24
Duodenum	30.31 ± 8.17	16.56 ± 8.68	22.38 ± 10.82	31.39 ± 22.29
Jejunum	55.07 ± 47.42	69.80 ± 20.76	45.88 ± 9.85	9.58 ± 7.36
Ileum	2.78 ± 1.23	13.31 ± 7.14	8.45 ± 10.81	8.89 ± 8.33
U-87 MG Tumour	3.56 ± 0.78	2.89 ± 0.53	0.89 ± 0.31	2.80 ± 0.70
SKOV-3 Tumour	3.47 ± 0.93	2.42 ± 0.40	0.73 ± 0.30	2.12 ± 0.56
Tumour-to-non-tumour ratios				
U87-MG to Blood	0.44 ± 0.13	0.77 ± 0.06	0.14 ± 0.04	0.83 ± 0.78
U87-MG to Muscle	2.45 ± 0.53	4.51 ± 0.58	0.90 ± 0.30	3.64 ± 1.55
SKOV-3 to Blood	0.43 ± 0.17	0.64 ± 0.18	0.12 ± 0.06	0.63 ± 0.51
SKOV-3 to Muscle	2.39 ± 1.22	3.78 ± 1.08	0.74 ± 0.45	2.76 ± 0.89

^*a*^Results are expressed as mean %ID/g at indicated uptake times ± SD (*n* ≥ 7)

### MicroPET imaging

**Fig. 5:**
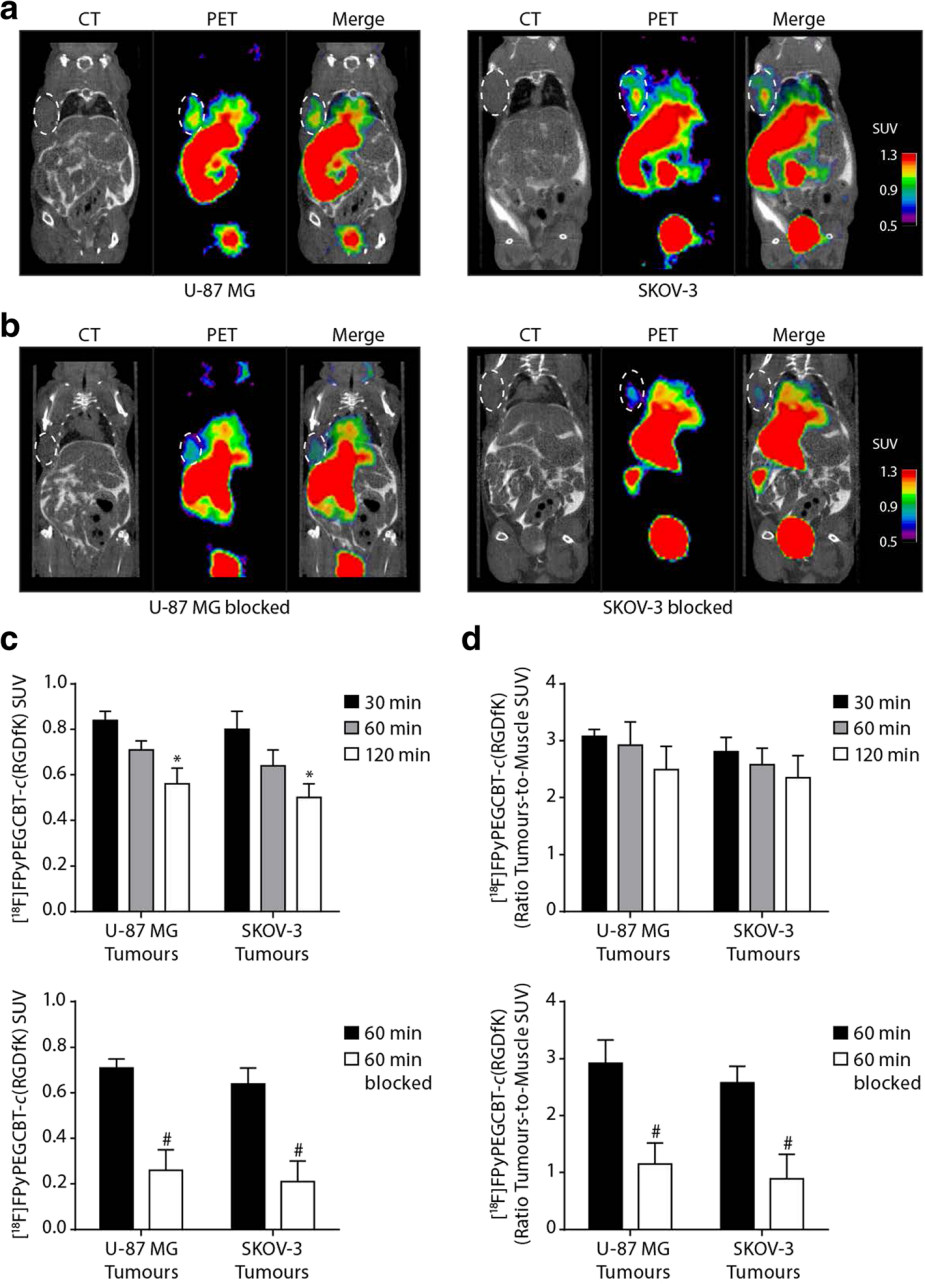
MicroPET/CT images of [^18^F]FPyPEGCBT-*c*(RGDfK) in nude mice bearing subcutaneous tumours. Coronal sections of representative microPET/CT images of nude mice bearing subcutaneous U-87 MG (**a**, *left*) and SKOV-3 (**a**, *right*) tumours after an uptake time of [^18^F]FPyPEGCBT-*c*(RGDfK) of 60 ± 10 min. Tumours are outlined by dashed circles. Similar experiments were performed with injections of a blocking *c*(RGDfV) peptide 5 min prior to the tracer (**b**). **c** Mean SUV of [^18^F]FPyPEGCBT-*c*(RGDfK) in U-87 MG and SKOV-3 tumours at indicated times (top graph) as well as in the 60 min-blocked experiment (*bottom graph*). **d** Tumours-to-blood and Tumours-to-muscle SUV ratios were also determined for the uptake time course of [^18^F]FPyPEGCBT-*c*(RGDfK) (*top graph*) and the blocked experiment (*bottom graph*). Data are means ± SD (*n* ≥ 5). Statistical differences were analysed with the one-way ANOVA test followed by Dunnett’s post hoc test as compared with 0.5 h uptake time (**c**, **d**, *top graphs*) or with non-blocked 1 h uptake time (**c**, **d**, *bottom graphs*); *p < 0.05, #*p* < 0.001

Micro-PET/CT analyses were performed on the same models as the ex vivo biodistribution. Mean SUVs and tumours-to-muscle SUV ratios were calculated after 30, 60 and 120 min (Fig. 5 and Additional file 1: Figure S3). U-87 MG and SKOV-3 tumours were evident 30 min after injection and the related SUVs decreased over time. The tumour-to-muscle ratios, ranging from 2.4 to 3.1 were not statistically different during the time course. Nevertheless, an obvious overall reduction of the accumulation of [^18^F]FPyPEGCBT-*c*(RGDfK) in non-targeted organs was more favourable for tumour visualisation at the 60 min timepoint. High tracer accumulation in organs responsible for the hepatobiliary excretion could be visualized, confirming the ex vivo biodistribution data. A blocking experiment with *c*(RGDfV) showed the specificity of the tracer uptake in U-87 MG and SKOV-3 tumours. Both SUVs and tumour-to-muscle ratios were diminished by a factor of 3, which is in accordance with the ex vivo biodistribution data.

## Discussion

The identification of non-invasive imaging tracers that can specifically target and quantify proteins controlling cancer progression is crucial for the development of personalised treatment. RGD-recognising integrins are promising targets, yet few radioligands targeting integrin receptors have been evaluated clinically to image angiogenesis and predict response to antiangiogenic therapy. We have recently synthesized a new RGD peptide, dubbed [^18^F]FPyPEGCBT-*c*(RGDfK), for the visualization of integrin expression by PET imaging. This radiotracer was prepared by condensation of a ^18^F-labelled cyanobenzothiazole (CBT) prosthetic group with a modified c(RGDfK) peptide (Inkster et al. 2015). The radiosynthesis relied on the high incorporation of ^18^F-fluoride onto 2-substituted pyridine precursor (Kuhnast et al. 2008; Inkster et al. 2008; Inkster et al. 2009) and the chemoselective ligation of CBT and 1,2-aminothiol moiety (Jeon et al. 2012). The prosthetic group and the peptide were both functionalized by a short polyethylene glycol chain to increase the hydrophilicity and bioavailability of the peptide tracer (Harris and Chess 2003; Wu et al. 2007a). The radiosynthesis of [^18^F]FPyPEGCBT-*c*(RGDfK) was achieved in 130 min, completely automated and required only one HPLC purification step, which was more practical than [^18^F]Galacto-*c*(RGDfK) (Haubner et al. 2004) but comparable to FPPRGD2 tracer (Liu et al. 2010). In this study we evaluated the preclinical biological properties of a [^18^F]FPyPEGCBT-*c*(RGDfK) in vitro, as well as in vivo, in a glioblastoma (U-87 MG) and an ovarian (SKOV-3) cancer model.

The binding properties of FPyPEGCBT-*c*(RGDfK) as assessed in a cell-binding assay demonstrated favourable albeit slightly lower IC_50_ values when compared to the reference *c*(RGDfV) peptide. A reduction of binding affinity upon insertion of a PEG modification has been previously reported (Chen et al. 2004a; Chen et al. 2004b). The binding properties of FPyPEGCBT-*c*(RGDfK) were in the same range than other potent monomeric *c*(RGD) derivatives like Galacto-*c*(RGDfK) (Liu et al. 2010). An increase of binding affinity of *c*(RGD) peptides is usually observed with a higher degree of multimerisation (Li et al. 2007). Indeed, IC_50_ measurements of the *c*(RGD) tetramer FPRGD4 (3.8 × 10^−8^ M) and the dimer FPPRGD2 (5.2 × 10^−8^ M) are an order of magnitude lower than the IC_50_ values of monomers like Galacto-*c*(RGDfK) (4.0 × 10^−7^ M) or FPyPEGCBT-*c*(RGDfK) (30.84 × 10^−7^ M) (Liu et al. 2010; Wu et al. 2007b). Nevertheless, the higher binding properties of FPRGD4 did not translate to improved results in vivo because of high background accumulation. Based on clinical studies with [^68^Ga]NOTA-*c*(RGDyK) (Jeong et al. 2008; Kim et al. 2012) and the dimers [^18^F]FRGD2 and [^18^F]FPPRGD2 (Chen et al. 2004a; Zhang et al. 2006; Wu et al. 2007a; Choyke 2011; Mittra et al. 2011; Liu et al. 2010), it seems that the most favourable conformations are the monomeric and dimeric scaffolds.

Further in vitro characterization of [^18^F]FPyPEGCBT-*c*(RGDfK) showed that 80 to 90 % of its internalisation was mediated by an integrin-related active transport in both cell lines studied. Furthermore, siRNA depletion experiments demonstrated that α_V_, β_3_ and β_5_ but not β_1_ subunits were responsible for [^18^F]FPyPEGCBT-*c*(RGDfK) uptake. Similarly, other *c*(RGD) peptides have been shown to specifically bind both α_V_β_3_ and α_V_β_5_ integrins by ELISA (Maschauer et al. 2014). Taken together, these results show that the α_V_β_3_-specificity claimed for many RGD peptide tracers cannot be concluded from competitive cell binding assay in the U-87 MG model. Promising peptide ligands with alternative binding motifs have recently been reported, including a) α_V_β_3_ but not α_V_β_5_ integrin (Zannetti et al. 2009); b) exclusively α_5_β_1_ (Neubauer et al. 2013; Haubner et al. 2014b); and c) exclusively α_V_β_6_ (Hausner et al. 2009). Such compounds have the potential to yield highly specific radiotracers.

Distribution of [^18^F]FPyPEGCBT-*c*(RGDfK) was assessed in mice bearing subcutaneous U-87 MG or SKOV-3 tumours by ex vivo analysis of selected tissues and microPET imaging. The specificity of ligand binding to RGD-recognising integrins was challenged by pre-injection of an excess of *c*(RGDfV). This demonstrated a significant reduction of the tumour uptake whereas no differences were observed in normal tissues except in the adrenals, which are known to express moderate levels of RGD-recognising integrins. The absolute tumour uptake of [^18^F]FPyPEGCBT-*c*(RGDfK) measured in the U-87 MG model ranged between 3.6 and 2.8 %ID/g over the 30 to 120 min time course; similar results were obtained in the SKOV-3 model. These uptake values were higher than those reported for the monomeric peptide tracer [^18^F]Galacto-*c*(RGDfK) (≈1.2 %ID/g in U-87 MG), and similar to dimeric *c*(RGD) tracer [^18^F]FPPRGD2 (≈2.8 %ID/g), but lower than [^18^F]CBTRGD2 (4.25 %ID/g) (Liu et al. 2010; Jeon et al. 2012). Tumour-to-muscles ratios of [^18^F]FPyPEGCBT-*c*(RGDfK) (≈4.0 measured by ex vivo biodistribution and ≈ 3.0 by PET imaging 1 h after injection) clearly allowed the visualisation of U-87 MG and SKOV-3 subcutaneous tumours by microPET imaging. These ratios are similar to [^18^F]CBTRGD2, but inferior to [^18^F]Galacto-*c*(RGDfK) or [^18^F]FPPRGD2 which exhibit ratios as high as 10 (Liu et al. 2010; Jeon et al. 2012). The relatively low tumour-to-background ratio of [^18^F]FPyPEGCBT-*c*(RGDfK) could be attributed to its prolonged presence in the blood, which resulted in its increased bioavailability but also to higher background accumulation. Moreover, the unwanted retention of convoluting radioactivity in the liver and the intestines suggests a significant degree of excretion via the hepatobiliary pathway. High excretion via this pathway rather than the renal route is widely thought to be correlated with the degree of lipophilicity of pharmaceuticals. It was anticipated that the inherent lipophilicity of CBT moiety might be counteracted by the two short PEG chains of [^18^F]FPyPEGCBT-*c*(RGDfK) (Jeon et al. 2012). Unfortunately, [^18^F]FPyPEGCBT-*c*(RGDfK) exhibits a higher lipophilicity than other potent *c*(RGD) peptide tracers (log D value of −1.2 vs. -2.3 to −3.2) (Haubner et al. 2004; Wu et al. 2007a; Inkster et al. 2015), which could account for its hepatobiliary excretion.

Taken all together, our results suggest that [^18^F]FPyPEGCBT-c(RGDfK) would require some design optimization to yield a clinical relevant radiolabeled RGD peptide for non-invasive angiogenesis imaging. At first, a dimer scaffold should be considered to increase the affinity towards the RGD-recognising integrin receptors. Then, to improve the biodistribution profile, the water solubility and lipophilicity have to be better balanced. It can effectively be achieved by: i) the introduction of a longer polyethylene glycol chain spacer, or ii) the presence of a positive charge, known to favour renal elimination, or iii) attachment of a chelating agent and subsequent labelling with a radiometal. Such structural modifications should lead to a tracer with improved pharmacokinetic/pharmacodynamics properties, namely a better accumulation of the tracer in the tumour, higher tumour-to-background ratios, lower uptake in non-target organs, and a faster clearance from the blood.

## Conclusion

[^18^F]FPyPEGCBT-*c*(RGDfK) demonstrated good specificity towards tumours expressing RGD-recognising integrins and was suitable for their detection in preclinical models. Indeed, this peptide tracer which binds to α_V_β_3_ and α_V_β_5_ integrins, showed a high stability in vivo and its favourable tumour uptake combined with a good tumour-to-background contrast was comparable to other reported RGD-based tracers. Nevertheless, its hepatobiliary excretion route could impair its general utility as a cancer-imaging agent. Because of the [^18^F]FPyPEGCBT-mediated radiolabeling approach is rapid and fully automated, it should prove useful for the ^18^F labelling of other biological targeting vectors which warrant clinical study, including *c*(RGDfK) multimers (Choyke 2011).

## Additional file

Additional file 1: Table S1. Statistical analyses of the biodistribution data of [^18^F]FPyPEGCBT-*c*(RGDfK) in nude mice bearing U-87 MG or SKOV-3 subcutaneous tumours^*a*^ *.* **Figure S1.** siRNA-mediated integrin depletions analysis by densitometry. αV, β1, β3 or β5 integrin subunits and indicated combinations were knocked-down by siRNA in U-87 MG and SKOV-3 and protein extracts were submitted to immunoblotting using specific antibodies. Expression levels of each integrin were then quantified with a Chemidoc MP. Data are mean fold expression changes in indicated samples as compared to untransfected cells (Unt) ± SD (*n* = 3). **Figure S2.** Uptake of [^125^I]echistatin in cells with selective siRNA-mediated integrin knocked down. U-87 MG and SKOV-3 cells knocked down for αV, β1, β3 or β5 integrin subunits and indicated combinations were incubated with 100 kBq/mL [^18^F]FPyPEGCBT-*c*(RGDfK) for 15 to 120 min at 37 °C. Depletions were verified by immunoblotting using specific antibodies as compared to untransfected cells (Unt) and to an irrelevant depletion of luciferase (si Luc). After incubation, cells were lysed and the cell-associated radioactivity was measured in a γ-counter. Data are means ± SD of at least three independent experiments performed in quadruplicate well. Statistical differences were analysed with the one-way ANOVA test followed by Dunnett’s post hoc test as compared to si Luc controls; #*p* < 0.001. **Figure S3.** MicroPET/CT images of [^18^F]FPyPEGCBT-*c*(RGDfK) in nude mice bearing subcutaneous tumours. Coronal sections of representative microPET/CT images of nude mice bearing subcutaneous U-87 MG (a) and SKOV-3 (b) tumours after indicated uptake times of [^18^F]FPyPEGCBT-*c*(RGDfK). Tumours are outlined by dashed circles. **Figure S4.** a, representative radio-TLC of mouse serum 2 h after injection of [^18^F]FPyPEGCBT-*c*(RGDfK). TLC eluent: 6:4 10 % aqueous ammonium acetate: MeOH. R_F_ = 0.8. b, Radio-TLC of [^18^F]FPyPEGCBT-*c*(RGDfK). TLC eluent: 6:4 10 % aqueous ammonium acetate: MeOH. R_F_ = 0.8. c, Radio-TLC of free [^18^F]F-. TLC eluent: 6:4 10 % aqueous ammonium acetate: MeOH. R_F_ = 0.3. (DOCX 2083 kb)
