# Supplementary material for: Preclinical validations of [18F]FPyPEGCBT-c(RGDfK): a 18F-labelled RGD peptide prepared by ligation of 2-cyanobenzothiazole and 1,2-aminothiol to image angiogenesis
Source: EJNMMI Radiopharm Chem. 2016 Oct 25;1:16. doi: 10.1186/s41181-016-0019-z (PMC5843817; doi:10.1186/s41181-016-0019-z)
Supplement: Supplementary file 1 — Statistical analyses of the biodistribution data of [18F]FPyPEGCBT-c(RGDfK) in nude mice bearing U-87 MG or SKOV-3 subcutaneous tumoursa . Figure S1. siRNA-mediated integrin depletions analysis by densitometry. αV, β1, β3 or β5 integrin subunits and indicated combinations were knocked-down by siRNA in U-87 MG and SKOV-3 and protein extracts were submitted to immunoblotting using specific antibodies. Expression levels of each integrin were then quantified with a Chemidoc MP. Data are mean fold expression changes in indicated samples as compared to untransfected cells (Unt) ± SD (n = 3). Figure S2. Uptake of [125I]echistatin in cells with selective siRNA-mediated integrin knocked down. U-87 MG and SKOV-3 cells knocked down for αV, β1, β3 or β5 integrin subunits and indicated combinations were incubated with 100 kBq/mL [18F]FPyPEGCBT-c(RGDfK) for 15 to 120 min at 37 °C. Depletions were verified by immunoblotting using specific antibodies as compared to untransfected cells (Unt) and to an irrelevant depletion of luciferase (si Luc). After incubation, cells were lysed and the cell-associated radioactivity was measured in a γ-counter. Data are means ± SD of at least three independent experiments performed in quadruplicate well. Statistical differences were analysed with the one-way ANOVA test followed by Dunnett’s post hoc test as compared to si Luc controls; #p < 0.001. Figure S3. MicroPET/CT images of [18F]FPyPEGCBT-c(RGDfK) in nude mice bearing subcutaneous tumours. Coronal sections of representative microPET/CT images of nude mice bearing subcutaneous U-87 MG (a) and SKOV-3 (b) tumours after indicated uptake times of [18F]FPyPEGCBT-c(RGDfK). Tumours are outlined by dashed circles. Figure S4. a, representative radio-TLC of mouse serum 2 h after injection of [18F]FPyPEGCBT-c(RGDfK). TLC eluent: 6:4 10 % aqueous ammonium acetate: MeOH. RF = 0.8. b, Radio-TLC of [18F]FPyPEGCBT-c(RGDfK). TLC eluent: 6:4 10 % aqueous ammonium acetate: MeOH. RF = 0.8. c, Radio-TLC of free [1 [file 41181_2016_19_MOESM1_ESM.docx]

**Additional file 1**

**Table S1 Statistical analyses of the biodistribution data of [^18^F]FPyPEGCBT-*c*(RGDfK) in nude mice bearing U-87 MG or SKOV-3 subcutaneous tumours*^a^***

**
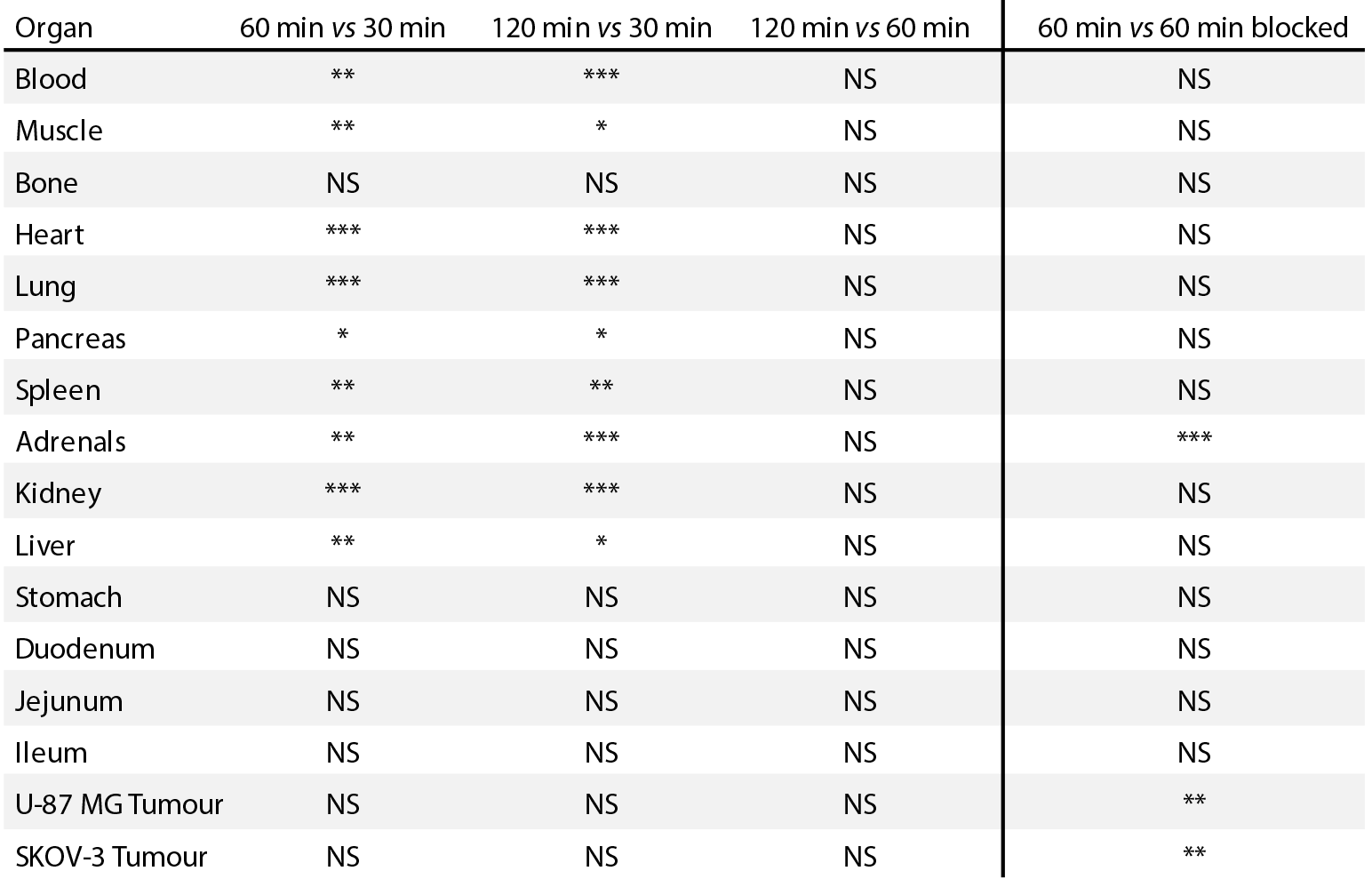
**

*^a^*Differences between %ID/g in organs at indicated uptake times (Table 1) were statistically assessed with the one-way ANOVA test followed by Tukey’s post hoc multiple comparisons test (n≥7); * p<0.05, ** p<0.01, *** p<0.001.


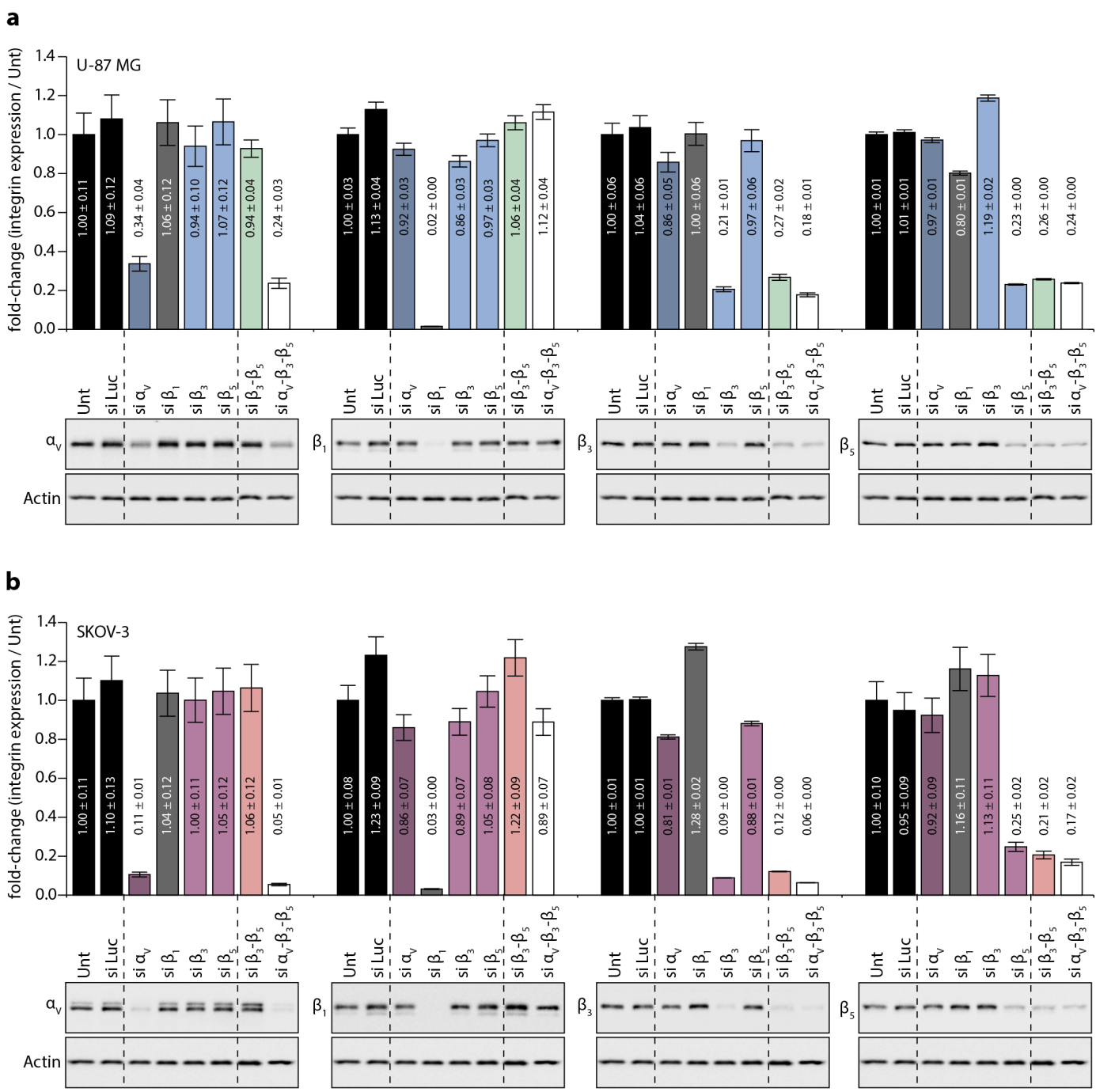


**Figure S1 siRNA-mediated integrin depletions analysis by densitometry.** α_V_, β_1_, β_3_ or β_5_ integrin subunits and indicated combinations were knocked-down by siRNA in U-87 MG and SKOV-3 and protein extracts were submitted to immunoblotting using specific antibodies. Expression levels of each integrin were then quantified with a Chemidoc MP. Data are mean fold expression changes in indicated samples as compared to untransfected cells (Unt) ± SD (n=3).


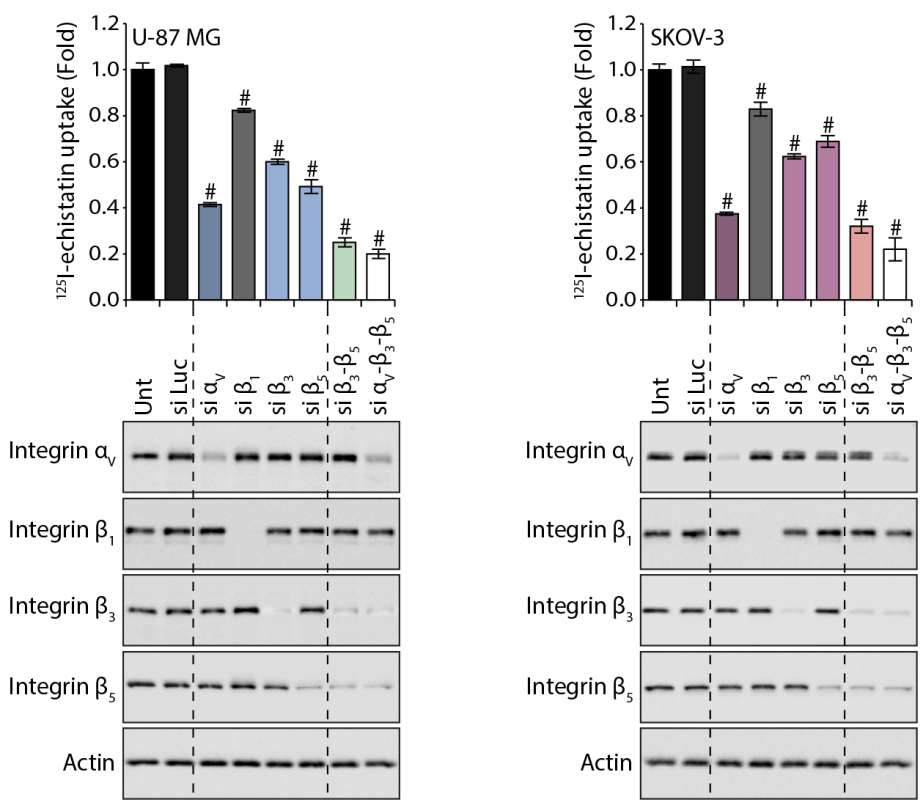


**Figure S2** **Uptake of [^125^I]echistatin in cells with selective siRNA-mediated integrin knocked down.** U-87 MG and SKOV-3 cells knocked down for α_V_, β_1_, β_3_ or β_5_ integrin subunits and indicated combinations were incubated with 100 kBq/mL [^18^F]FPyPEGCBT-*c*(RGDfK) for 15 to 120 min at 37°C. Depletions were verified by immunoblotting using specific antibodies as compared to untransfected cells (Unt) and to an irrelevant depletion of luciferase (si Luc). After incubation, cells were lysed and the cell-associated radioactivity was measured in a γ-counter. Data are means ± SD of at least three independent experiments performed in quadruplicate well. Statistical differences were analysed with the one-way ANOVA test followed by Dunnett’s post hoc test as compared to si Luc controls; # p<0.001.


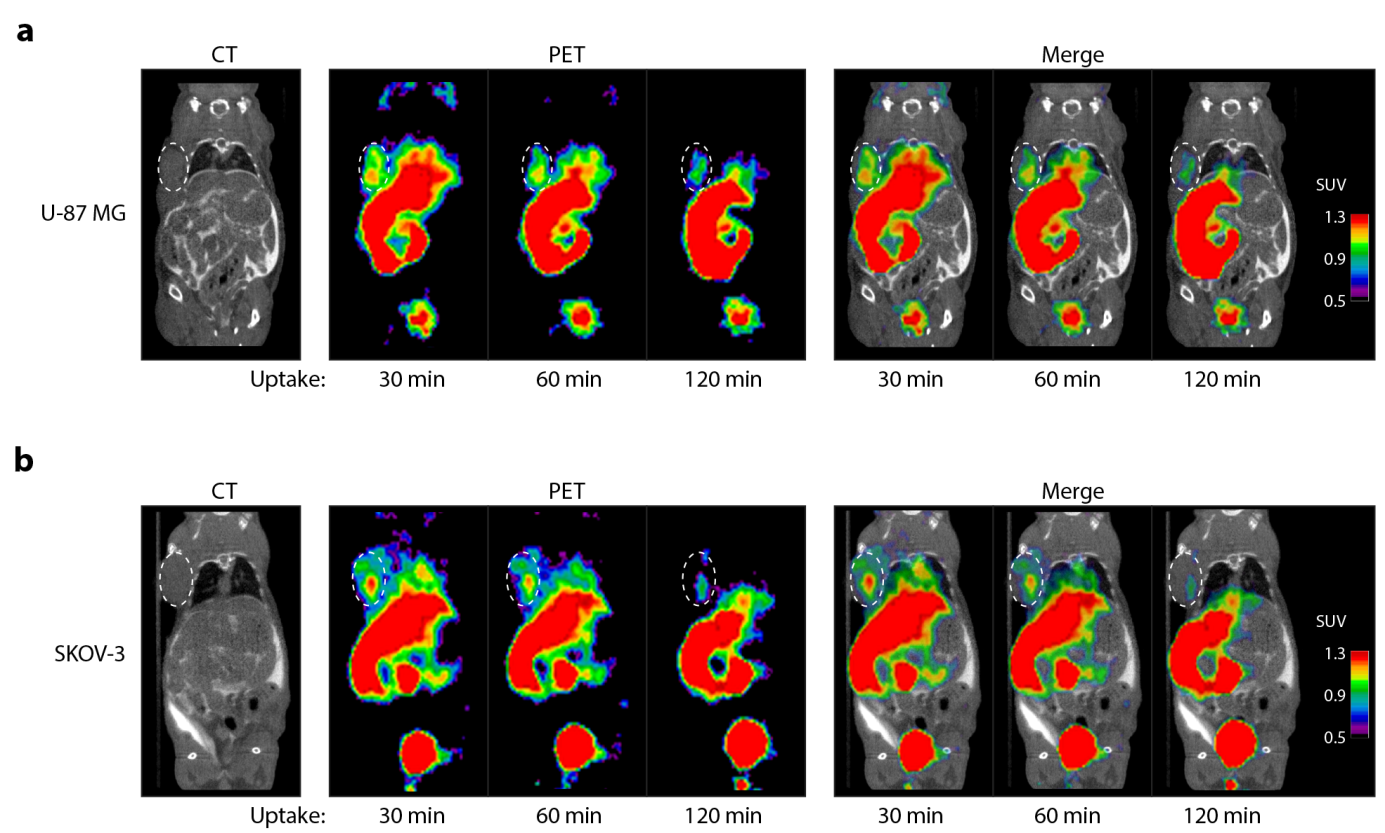


**Figure S3 MicroPET/CT images of [^18^F]FPyPEGCBT-*c*(RGDfK) in nude mice bearing subcutaneous tumours.** Coronal sections of representative microPET/CT images of nude mice bearing subcutaneous U-87 MG (**a**) and SKOV-3 (**b**) tumours after indicated uptake times of [^18^F]FPyPEGCBT-*c*(RGDfK). Tumours are outlined by dashed circles.


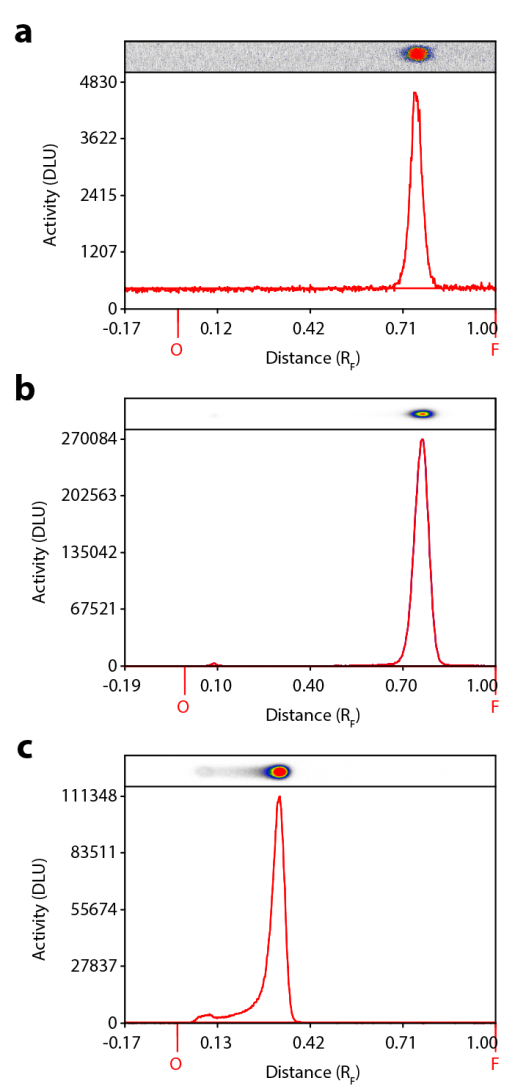


Figure S4 a, representative radio-TLC of mouse serum 2 h after injection of [^18^F]FPyPEGCBT-*c*(RGDfK). TLC eluent: 6:4 10% aqueous ammonium acetate: MeOH. R_F_ = 0.8. b, Radio-TLC of [^18^F]FPyPEGCBT-*c*(RGDfK). TLC eluent: 6:4 10% aqueous ammonium acetate: MeOH. R_F_ = 0.8. c, Radio-TLC of free [^18^F]F^-^. TLC eluent: 6:4 10% aqueous ammonium acetate: MeOH. R_F_ = 0.3.
